# Efficient CRISPR-Cas9 Gene Disruption System in Edible-Medicinal Mushroom *Cordyceps militaris*

**DOI:** 10.3389/fmicb.2018.01157

**Published:** 2018-06-12

**Authors:** Bai-Xiong Chen, Tao Wei, Zhi-Wei Ye, Fan Yun, Lin-Zhi Kang, Hong-Biao Tang, Li-Qiong Guo, Jun-Fang Lin

**Affiliations:** ^1^Department of Bioengineering, South China Agricultural University, Guangzhou, China; ^2^Research Center for Micro-Ecological Agent Engineering and Technology of Guangdong Province, Guangzhou, China; ^3^Guangzhou Alchemy Biotechnology Co., Ltd., Guangzhou, China

**Keywords:** *Cordyceps militaris*, CRISPR-Cas9, gene disruption, edible-medicinal mushroom, fungi industry

## Abstract

*Cordyceps militaris* is a well-known edible medicinal mushroom in East Asia that contains abundant and diverse bioactive compounds. Since traditional genome editing systems in *C. militaris* were inefficient and complicated, here, we show that the codon-optimized *cas9*, which was used with the newly reported promoter Pcmlsm3 and terminator Tcmura3, was expressed. Furthermore, with the help of the negative selection marker *ura3*, a CRISPR-Cas9 system that included the Cas9 DNA endonuclease, RNA presynthesized *in vitro* and a single-strand DNA template efficiently generated site-specific deletion and insertion. This is the first report of a CRISPR-Cas9 system in *C. militaris*, and it could accelerate the genome reconstruction of *C. militaris* to meet the need for rapid development in the fungi industry.

## Introduction

*Cordyceps militaris* is a kind of ascomycetous (Wang et al., 2008; Cui, 2015) farming mushroom with multiple medicinal (Reis et al., 2013) uses. Bioactive compounds, such as cordycepin (Tuli et al., 2015), ergosterol, ergothioneine (Cohen et al., 2014), cordyceps polysaccharides (Zhang et al., 2015), isolated from *C. militaris* have been demonstrated to have antitumorigenic (Wu et al., 2007), anti-inflammatory (Noh et al., 2008; Taofi et al., 2016), antioxidant (Kim et al., 2006) and antimicrobial functions (Zhou et al., 2016). Additionally, effective components in extracts could be useful materials in skin cosmetics (Chien et al., 2008). Therefore, due to these multiple applications, *C. militaris* has been regarded as a potential industrial mushroom in recent years.

To classify the synthetic mechanism of the bioactive compounds in *C. militaris*, various metabolic pathways were predicted (Zheng P. et al., 2011) by high-throughput sequencing. To further construct engineered strains with better productivity, the establishment of an efficient genome-editing method is vital to the complete process. However, reports of genomic editing in *Cordyceps* are still rare (Zheng Z. et al., 2011; Yang et al., 2016). Traditional genomic editing technologies such as methods based on homologous recombination (HR) and *Agrobacterium*-mediated random gene insertion were insufficient and too complicated to satisfy the requirement of accurate and repeatable editing.

Clustered regularly interspaced short palindromic repeats (CRISPR) system was a newly innovative and efficient genome-editing tool with gene knockout, insertion and replacement abilities. Among all types of CRISPR systems, the type II CRISPR system, which consists of only two compounds, the CRISPR-associated endonuclease Cas9 from *Streptococcus pyogenes* and the single-guide RNA (sgRNA), which is fused with precrRNA and tracrRNA (Cong et al., 2013), is the most popular one. With guidance to specific sites by the sgRNA, genome sequences are cut, and double-stranded breaks (DEGs) are generated by Cas9. Subsequently, gene knock-out would be caused by non-homologous end-joining (NHEJ) while gene replacement would be obtained via HR. Among the vast applications of the type II CRISPR system in a wide range of species, the CRISPR-Cas9 system is also implemented in filamentous fungi such as *Aspergillus nidulans* (Katayama et al., 2015; Nødvig et al., 2015), *Trichoderma reesei* (Liu et al., 2015) and *Neurospora crassa* (Matsu-ura et al., 2015). However, different from these filamentous fungi, industrial mushrooms such as those in the genus *Cordyceps*, possess a much more complicated two-stage growth and a well-known high ratio of fruiting body gemmated degeneration, which leads to more difficulties in the application of a CRISPR system.

In this study, the application of a CRISPR system in the genus *Cordyceps* is reported for the first time. With the help of the newly discovered promotor Pcmlsm3 and terminator Tcmura3, the *cmcas9* gene was successfully transformed into the cells of *Cordyceps militaris*. A stable Cas9-expressing strain was proved by a fluorescent GFP tag and western blot analysis. sgRNA and donor single-stranded DNA (ssDNA) synthesized *in vitro* were further transformed into the *Cordyceps militaris* strain with *cmcas9* expression. As a result, the target gene *ura3*, which was first used in *S. cerevisiae* as a selective marker (Boeke et al., 1984), was successfully disrupted by the CRISPR-Cas9 system.

## Materials and methods

### Strains and cultural conditions

*Escherichia coli* strain DH5α (Weidi Bio, Shanghai, China) was used for vector construction. *Agrobacterium tumefaciens* AGL-1 (Weidi Bio, Shanghai, China) and the pCAMBIA0390 plasmid (Cambia, Queensland, Australia) were used for mediating the fungal transformation. *C. militaris* CM10 was purchased from Lucky Mushroom Garden (http://www.bjjixunyuan.com, Beijing, China) as the host for gene disruption. *C. militaris* CM10 was cultured in potato peptone dextrose agar (PDA) at 25°C. The materials for the nucleic acids are shown in Table 1.

**Table 1:** Materials of strains and nucleic acid.

**Strains**	**Source**
*Escherichia coli* strain DH5α	Purchase from Weidi Bio, Shanghai, China
*Agrobacterium tumefaciens* AGL-1	Purchase from Weidi Bio, Shanghai, China
*Cordyceps militaris* CM10	Purchase from Lucky Mushroom Garden (http://www.bjjixunyuan.com), Beijing, China
*C. militaris* C9	This study, *C. militaris:*: *blpR*-*cmcas9*-*gfp*
**VECTORS**	
pMD18T	Purchase from Takara, Beijing, China
pCAMBIA03301	Purchase from Cambia, Queensland, Australia
pCAMBIA0390	Purchase from Cambia, Queensland, Australia
cmgpd + T, cmlsm3 + T, cmura3 + T	This study, Table 2, Figure 1
p390cm-gpd-c9gm	This study, Table 2, Figure 1
PGH-gfp, PGH-CmCas9	This study, synthesized by GENEray, Shanghai, China
p390-blpR-cmcas9	This study, Table 2, Figure 1
p390-blpR-gRNA-cmcas9	
**DNAS**	
Lcmlsm3-blp-Tcmura3	This study, Table 2, Figure 1
ortUra3-1	This study, Table S4, Figure 5B
**RNAS**	
sgRNA-gUra3-1	This study, Table S5, Figure 5
sgRNA-gUra3-2	This study, Table S5, Figure 5
**Genes**	**Description**
*cmcas9*	From *S. pyogenes* Cas-9 but codon-optimized for *C. militaris*
*gfp*	Green fluorescent protein eGFP
*myc*	Nuclear localization signal c-Myc (PAAKRVKLD)
*cmgpd*	Glycerol-3-phosphate dehydrogenase of *C. militaris* (Accession No. NW_006271970.1)
*cmlsm3*	U6 small nuclear ribonucleoprotein of *C. militaris* (Accession No. NW_006271974.1)
*cmura3*	Orotidine 5′-phosphate decarboxylase of *C. militaris* (Accession No. NW_006271972.1)
*blpR*	Phosphinothricin acetyltransferase

### Construction of plasmids

**Figure 1:**
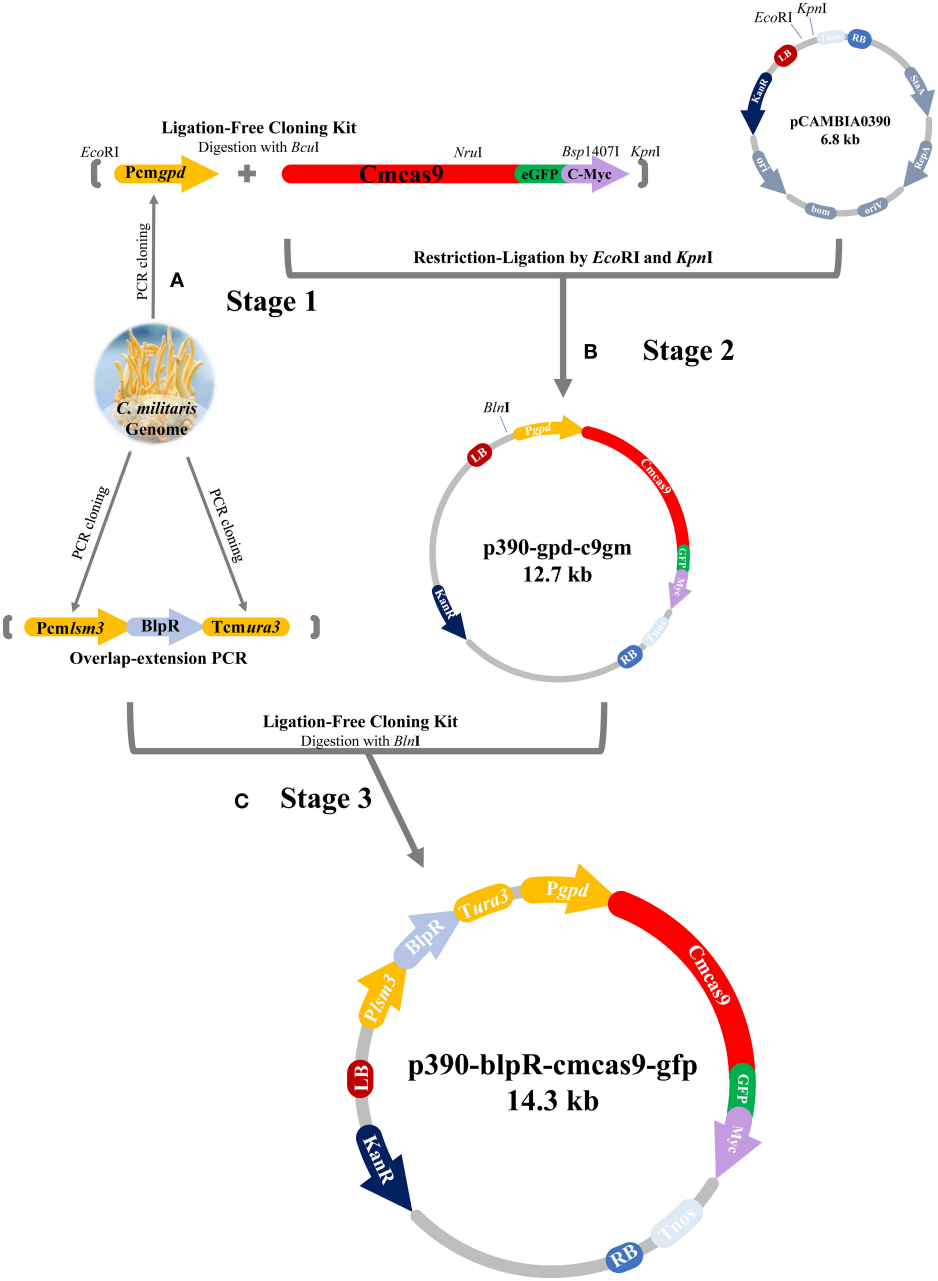
Flow diagram of CRISPR-Cas9 vector construction. **(A)** Pcm*gpd*, Pcm*lsm3* and Tcm*ura3* were cloned from the genome of *C. militaris*. **(B)** The cassette P*cmgpd*-*cmcas9*-*gfp*-*myc* was ligated into pCAMBIA0390 to build the p390cm-gpd-c9gm vector. **(C)** The cassette P*cmlsm3*-*blpR*-T*cmura3* was ligated into p390cm-gpd-c9gm to build p390-blpR-cmcas9-gfp.

The *cas9* gene, in which a codon from *Streptococcus pyogenes* was optimized for *C. militaris* and was then renamed as *cmcas9* (*cmcas9*, Table S1, Accession No. MG736726), was synthesized and ligated into the vector PGH (Shanghai GENEray Biotech Co., Ltd., Shanghai, China). The *gfp* gene, which was synthesized and cloned from the plasmid PGH-gfp (Shanghai GENEray Biotech Co., Ltd., Shanghai, China) and followed by a c-Myc nuclear localization signal (NLS) (PAAKRVKLD) which is more effective than the typically used SV40 NLS (Ray et al., 2015), was ligated to the C-terminal of *cmcas9* using a Ligation-Free Cloning kit (ABM, Vancouver, Canada). Driven by a constitutive promotor Pcmgpd (Gong et al., 2009), the cassette *cmcas9*-*gfp*-*myc*, which was cloned from the native promoter (Accession No. FJ374269) of glycerol-3-phosphate dehydrogenase (*gpd*) in *C. militaris* by the primer pair PgpdF/PgpdR, was ligated into pCAMBIA0390 by *Eco*RI/*Kpn*I to build the p390cm-gpd-c9gm vector. For the construction of the marker cassette, the promotor (Table S2) of the U6 small nuclear ribonucleoprotein (*lsm3*) in *C. militaris* (NW_006271974.1) and the terminator (Table S3) of orotidine 5′-phosphate decarboxylase (*ura3*) in *C. militaris* (NW_006271972.1) were cloned by primers Plsm3F/Plsm3R and ura3F/ura3R, respectively. The phosphinothricin acetyltransferase gene *blpR* was used as a positive selection marker for resistance to the toxic effect of Basta (glufosinate-ammonium) (XB-BIO, Guangzhou, China); it was cloned from pCAMBIA3301 (Cambia, Queensland, Australia) and driven by the Pcmlsm3 promoter and the Tcmura3 terminator. Lastly, the Pcmlam3-*blpR*-Tcmura3 cassette was ligated into the p390cm-gpd-c9gm vector to generate the *blpR* and *cmcas9* expression vector p390-blpR-cmcas9-gfp (Figure 1, Table 2, Accession No. MG736726).

**Table 2:** Methods and primers for vectors construction.

**Templates**	**Methods**	**Products**	**Primers**	**Sequences (5′−3′)**
*C. militaris* genome DNA	PCR cloning, pMT18T (Takara, Beijing, China)	cmgpd + T	PgpdF	GGAGGACGCTGAATGAGGACCAAGA
			PgpdR	TTCGCTTTACCAACCAAACCATAACCTG
		cmlsm3 + T	Plsm3F	AGGAACGGGTCCAGGCAGCGTATGA
			Plsm3R	CGCAACGGCAAGGAGATGTAAACCA
		cmura3 + T	PTura3F	CATTCCCAACGTTAGCCCATTAACCAG
			PTura3R	GGTGGCATTCGAAACAGGATATGACG
cmgpd + T	Ligation-Free Cloning Kit, *Nru*I*, Bsp*1407I*, Bcu*I*, Eco*RI*, Kpn*I.	p390cm-gpd-c9gm	aPGH-gpd	TTCCCAGATCCCATGAATTCGGCCAATTTGTCTAT
			agpd-C9	TCTTGTCCATACTAGTTGTTCTTGATTAGAA
PGH-gfp			aPGH-Myc-gfp	GTGATATCCCTGTACGGTACCTTAGTCAAGCTTGA
			gfp	CACGCTTGGCGGCAGGCTTGTACAGCTCGTC
			aC9-gfp	GCGGTGACGAGGGTGCCGTGAGCAAGGGCGAGG
PGH-CmCas9			aNru-C9	GCTCGTGGCAACTCGCGATTCGCTTGGATGA
			aC9-EGA	GGCACCCTCGTCACCGCCCAGC
pCAMBIA0390				
cmlsm3 + T	Overlap-extension PCR	line-Pcmlsm3-blp-Tcmura3	a390-lsm3	GAATTAATTCCCTAGTGTCTTTTCGCGCGGCG
			flsmR	TGGGCTCATGGATCCTATGAAATTATGAGCGCT
pCAMBIA3301			fTu3-blp	GACGAAGTGGTGCCATCAAATCTCGGTGAC
			fblp	GGATCCATGAGCCCAGAACGACGC
cmura3 + T			fblp-Tu3	GTCACCGAGATTTGATGGCACCACTTCGTC
			a390-Tu3	AACATGGTGGCCTAGGTCAAAAAGGTTATCAGGGACG
p390cm-gpd-c9gm	Ligation-Free Cloning Kit, *Bln*I	p390-blpR-cmcas9-gfp (Accession No. MG736726)		
Lcmlsm3-blp-Tcmura3				

### *Agrobacterium*-mediated transformation

The *Agrobacterium tumefaciens*-mediated transformation (ATMT) was used as described in a previously study (Zheng Z. et al., 2011) with some modification. To identify the resistance marker for detecting the transformants of ATMT, conidia of *C. militaris* were harvested and inoculated onto PDA with different Basta, G418, phleomycin, hygromycin or nourseothricin concentrations (100, 200, 300, 400, 500, 1,000, 2,000, 3,000, and 4,000 μg ml^−1^; Lou et al., 2018). In the process of transformation, *A. tumefaciens* AGL-1 carrying the vector p390-blpR-cmcas9 was grown in LB or IM (Zheng Z. et al., 2011 with 200 mM acetosyringone; Sigma-Aldrich, MO, USA) medium with kanamycin (50 μg ml^−1^) and carbenicillin (50 μg ml^−1^) on a shaker (180 rpm, 28°C). Once the cell density (OD_600_) reached 0.8, *A. tumefaciens* was mixed with *C. militaris* conidia suspensions (1 × 10^4^ conidia ml^−1^) in equal volumes (50 μl) and inoculated onto IMA (with 200 mM AS, pH 5.5) at 25°C for 3 days. Subsequently, cellophane sheets were transferred to potato PDA with cefotaxime (100 μg ml^−1^) and the appropriate drugs to kill *Agrobacterium* and wild-type *Cordyceps*. After 5 days of cultivation, individual mycelia were tested by PCR verification using primer pairs of Cas9F/R-491 (Table 3). Then, the putative transformants were tested by quantitative real-time PCR, western blot and fluorescence microscopy. Light and fluorescence microscopy were performed with a Zeiss Observe.A1 Axio microscope (Carl Zeiss AG, Oberkochen, Germany) equipped with a 40 × objective, a ZEISS Axiocam 503 camera (Carl Zeiss AG, Oberkochen, Germany), and the appropriate filter (excitation, 395–440 nm, and emission, 470 nm).

**Table 3:** Primers for PCR and qPCR.

**Usages**	**Targets**	**Primers**	**Sequences (5′-3′)**
qPCR analysis	*tef1*	qTefF	GTCAAGGAAATCCGTCGTGGTAA
		qTefR	GCAGGCGATGTGAGCAGTGTG
	*cmcas9*	qCasF-126	AGAAGGGTAACGAGCTTGCC
		qCasR-126	TGTGCTGCTCAACGAAAAGC
	gRNA	qRnaF-140	ATGAGTCCGTGAGGACGAAAC
		qRnaR-140	GCCAAAAGCACCACCGACTC
PCR varification	cmcas9	Cas9F-491	GTCTGAGTTCGTTTACGGTGACTACAA
		Cas9R-491	TGATGGTGATACCCAGGAGTTCTTT
	cmura3	Ura3F	ATGGTGGCTCCTCACCCTACTCTCAAG
		Ura3R	TCACCGCACTCTCTCCGTGTATGCT

### Quantitative real-time PCR (qPCR)

Total RNA was extracted from 100 mg of frozen mycelia pellets using an E.Z.N.A. Fungal RNA Miniprep kit (OMEGA Bio-Tek Inc., GA, USA). The qPCR template cDNA was synthesized from 1 μg of total RNA by using TransScript All-in-One First-Strand cDNA Synthesis SuperMix for qPCR (TransGen Biotech, Beijing, China). All primers used for quantitative real-time PCR (qPCR) are listed in Table 3. Each qPCR system consisted of a total volume of 20 μl, which contained 50 ng of cDNA, 160 nM each of the relevant primers and TransStart Tip Green qPCR SuperMix (TransGen Biotech). All qPCR was carried out by a QuantStudio 3 Real-Time PCR System (Thermo Fisher Scientific, MA, USA), following the reaction parameters from the TransStart Tip Green qPCR SuperMix instruction book (TransGen Biotech). Using the elongation factor 1-alpha (*tef1*) gene (NW_006271969.1) as an internal control for each sample (Lian et al., 2014), the relative mRNA levels were calculated by the delta-delta Ct method (Livak and Schmittgen, 2001).

### Western blot

Protein was extracted from the mycelia by radioimmunoprecipitation assay (RIPA) buffer (Sigma-Aldrich, USA) for western blotting. The supernatants were collected after centrifugation at 10,000 × g for 5 mins and mixed with 5 × loading buffer (60 mmol/L Tris–HCl, 2% SDS, 25% glycerol, 5% 2-mercaptoethanol, and 0.2% bromophenol blue) at a ratio of 1:4. Samples were then heated at 95°C for 5 min and separated on 5% SDS-polyacrylamide gel. The separated proteins were then transferred to a 0.22 μm polyvinylidene difluoride (PVDF) membrane (Perkin Elmer, MA, USA). The primary antibody used for western blotting was Guide-it™ Cas9 Monoclonal Antibody (Takara, Beijing, China). The secondary antibody was HRP-conjugated Goat Anti-Mouse Secondary Antibody (ABmart, Shanghai, China).

### *In vitro* synthesis of sgRNA and ssDNA

To evaluate the efficiency of the CRISPR-Cas9 system in *C. militaris*, the orotidine 5′-phosphate decarboxylase gene, *ura3*, in *C. militaris* (Accession No. NW_006271972.1) was chosen as the editing target. The sequence of the *ura3* gene and the *C. militaris* genome were uploaded into the Eukaryotic Pathogen gRNA Design Tool (Peng and Tarleton, 2015) (EuPaGDT; available at http://grna.ctegd.uga.edu/) to design a single-stranded DNA (ssDNA) template for target sites and primers for related sgRNA (Kistler et al., 2015). Two target sites (called gUra3-1 and gUra3-2; **Figure 4A**) were chosen, and one oligonucleotide repair template for gUra3-1(called ortUra3-1; Figure 4B) was synthesized, which was complementary to the gUra3-1 target site with 30-bp homology arms around the inserted sequence (TAGATAGATAG) on each side (Table S4). sgRNA for gUra3-1 and gUra3-2 were subsequently synthesized (called sgRNA-gUra3-1 and sgRNA-gUra3-2, Table S5) *in vitro* by a T7 High Efficiency Transcription kit (TransGen Biotech, Beijing, China).

### PEG-mediated protoplast transformation

The PEG-mediated protoplast transformation was modified as previously described (Binningerl et al., 1987). To identify the resistance marker for detecting the transformants with target site mutations, mycelia of *C. militaris* were inoculated onto PDA with different 5-fluoroorotic acid (5-FOA) or 5-fluorocytosine concentrations (25, 50, 75, 100, 150, 200, and 300 μg ml^−1^). *C. militaris* mycelia were cultured in 100 ml of potato dextrose broth medium at 25°C without shaking and then harvested by centrifugation after 7 days. Using sterile water and 0.7 M KCl to wash twice, the mycelia were digested by 0.7 M KCl with 2% lywallzyme (Guangdong Culture Collection Center, Guangzhou, China) at 30°C with gentle shaking (85 rpm min^−1^) for 3 h. Protoplasts were filtered and washed in 0.7 M KCl twice. After subsequent washing in STC buffer (1 M sorbitol, 25 mM CaCl_2_, 10 mM Tris-Cl pH 7.5), the protoplasts were resuspended in STC buffer at a density of 10^8^ protoplasts in each milliliter. Each 50 μl of protoplasts were mixed with 2 μg of sgRNA or/and 3 μg of ssDNA and then placed on ice for 5 min. A volume of 500 μL of PEG buffer (PEG 4000 25%, 25 mM CaCl_2_, 10 mM Tris-Cl pH 7.5) was slowly added. The whole mixture was incubated at room temperature for 20 min and then mixed with 500 μl of STC buffer and incubated at room temperature for 5 min. Protoplasts were harvested by centrifugation, resuspended in STC buffer and then incubated in PDA with 0.8 M mannitol at 25°C. After 3 days of regeneration, PDA medium with 5-FOA or 5-fluorocytosine but only half agar was used to directly cover the surface. Until the mycelia had grown out of the medium, individuals were picked and tested by PCR verification using the primer pair Ura3F/R (Table 3).

## Results

### Identification of resistance marker for *C. militaris*

To identify the resistance marker for genetic manipulation, hygromycin was primarily used as a selection agent according to a previous study in *C. militaris* (Mullins et al., 2001). However, hygromycin did not have a toxic effect on CM10 or the other *C. militaris* strains in our purchase stock (testing dose up to 4 g L^−1^). Several other drugs, such as G418, phleomycin, nourseothricin, Basta, 5-fluorocytosine and 5-FOA, that are commonly used in the genetic manipulation of fungi were tested. Only Basta and 5-FOA showed obvious lethality to the conidia or mycelia of *C. militaris* CM10. The effect of the minimum inhibitory concentration of Basta and 5-FOA on CM10 was determined by cultivating CM10 on PDA plates with different drug concentrations for a month. The growth of conidia was completely inhibited at a Basta concentration of 0.4 g L^−1^, and the growth of mycelia was completely inhibited at a 5-FOA concentration of 0.1 g L^−1^ within a month. Therefore, the resistance genes for Basta (*blpR*) and 5-FOA were chosen for further study. The Basta resistance gene *blpR* was ligated into p390-cmcas9 for positive selection in the construction of a genome-editing system.

### Cotransformation of a single vector with the sgRNA-cm*cas9* cassette

**Figure 2:**
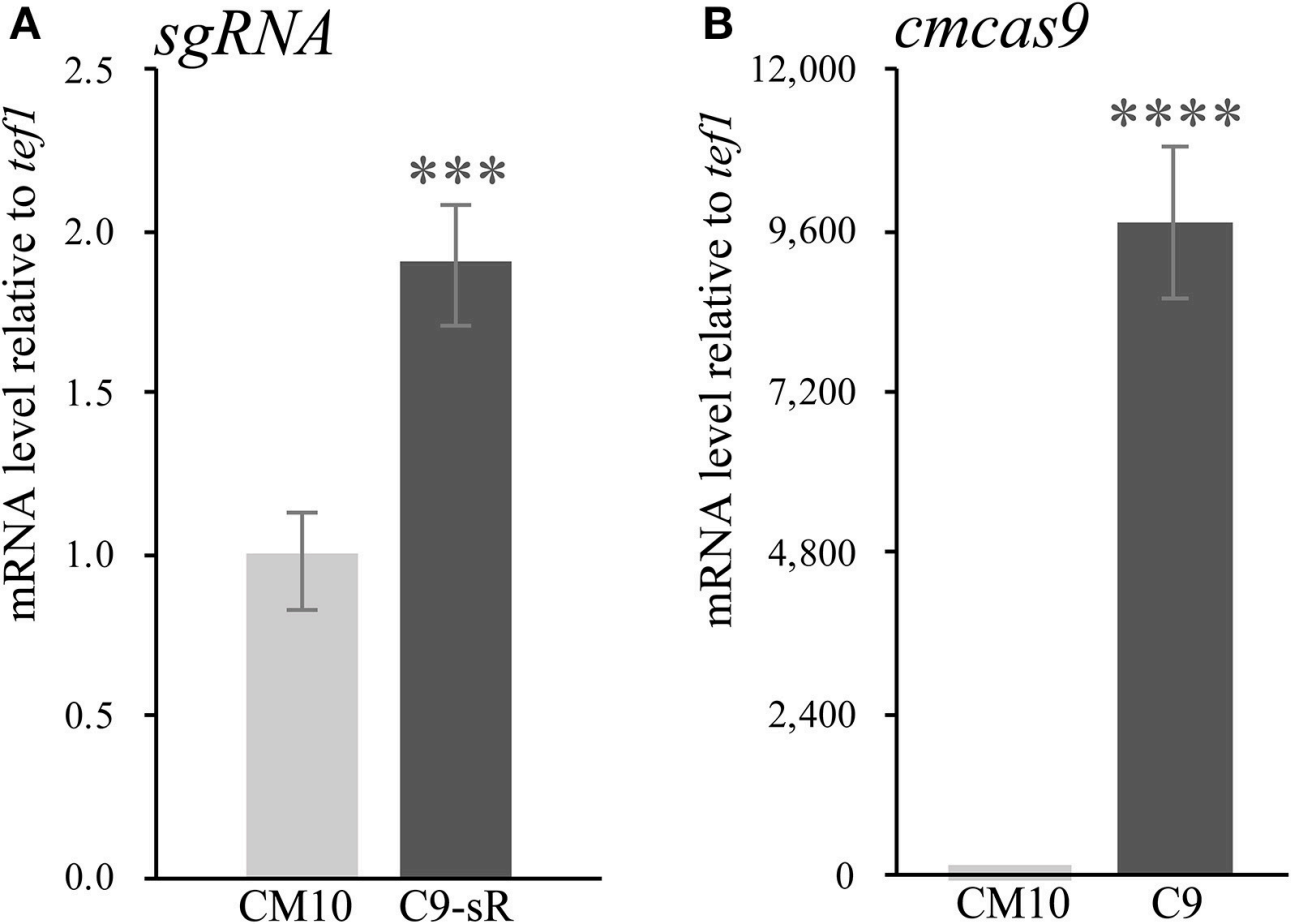
The qPCR experiments were carried out in biological triplicate. Data are represented as the mean ± SEM (*n* = 9). Statistical analyses were performed using *t*-tests compared with wild-type “CM10” (^***^p < 0.001, ^****^p < 0.0001). **(A)** The expression level of sgRNA in the transformant *C. militaris* C9-sR (*C. militaris:*: *blpR*-*cmcas9*-*gfp-HH-sgRNA-HDV*). **(B)** The expression level of Cas9 in the transformant *C. militaris* C9 (*C. militaris:*: *blpR*-*cmcas9*-*gfp*).

Since there were only a few available resistance markers in *C. militaris*, sgRNA and the *cas9* gene were primarily ligated and expressed as a cassette. Since identification of the RNA polymerase III promoter in *C. militaris* failed, a composite RNA molecule with an sgRNA scaffold, hammerhead (HH) and hepatitis delta virus (HDV)-type ribozymes, which was predicted to process in a self-catalyzed manner and then to release the sgRNA from a larger transcript as previously reported (Gao and Zhao, 2014), was adopted with an RNA polymerase II promoter and terminator from *Aspergillus nidulans trpC* (Zheng Z. et al., 2011) for the synthesis of sgRNA. These molecular elements were all constructed into p390-blpR-cmcas9-gfp to build p390-blpR-sgRNA-cmcas9-gfp (the sequence of the sgRNA cassette is shown in Table S6) and cotransformed into *C. militaris* CM10 subsequently. After resistance detection, PCR amplification and gene sequencing of *ura3*, more than 100 transformants with *cmcas9* gene integration were obtained. Nevertheless, there were no strains with mutations in the *ura3* gene identified in all these transformants. Then, a putative transformant was picked randomly to verify the transcript of the sgRNA by qPCR (Figure 2A). The sgRNA cassette had been transcribed, but it seems that it failed to work cooperatively with Cas9.

### Stable expression of the *cas9* gene in *C. militaris*

**Figure 3:**
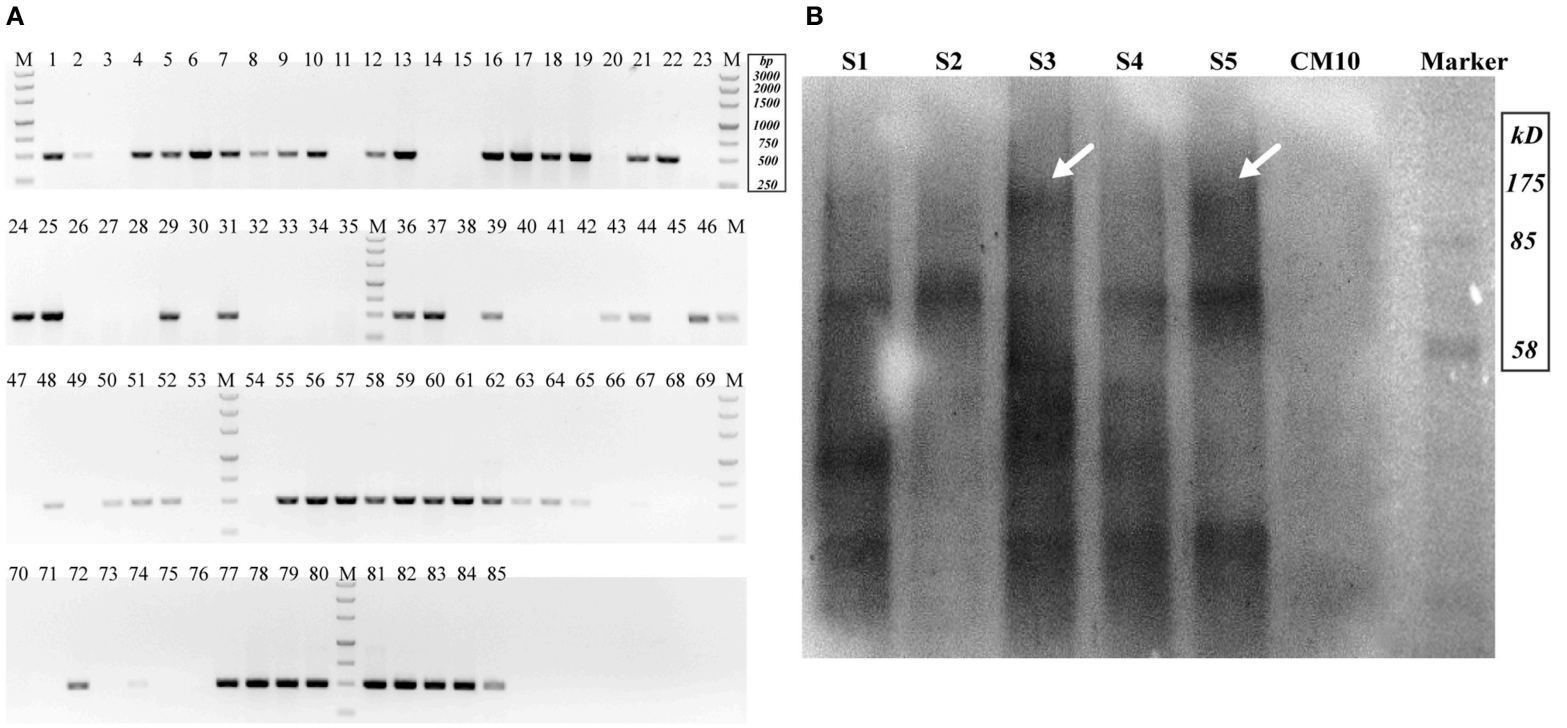
Detection of Cas9-expressing transformants. **(A)** Nucleic acid gel electrophoresis analysis of putative Cas9 transformants (Expected target band was 491 bp). **(B)** Western blot for Cas9 detection (S1–S5 represented putative transformants; CM10 represented the negative control; the expected Cas9 band was 186.5 kDa and is indicated by arrows).

**Figure 4:**
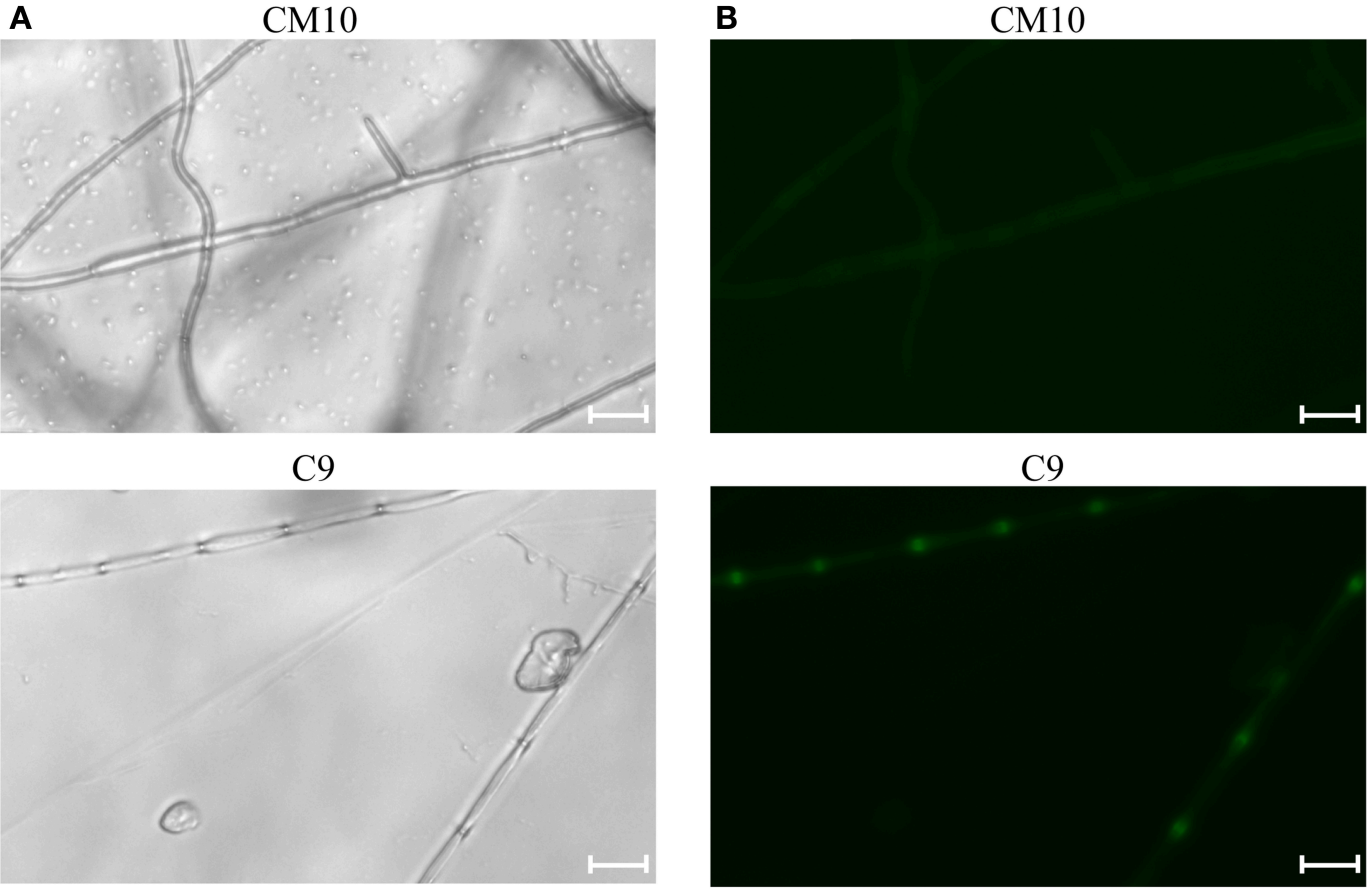
Mycelia of the Cas9-GFP transformant C9 and negative control CM10 were shown under **(A)** bright field image and **(B)** fluorescence microscope (excitation, 395 to 440 nm, and emission, 470 nm). The bars were 20 μm.

Cotransformation of the sgRNA-*cas9* cassette failed to function in *C. militaris*. To classify the failure of the sgRNA-*cas9* cassette, stable expression of the *cas9* gene became the priority target. After the construction and transformation of expression vector p390-blpR-cmcas9-gfp, 85 transformant strains were harvested using herbicide Basta (400 μg ml^−1^) as a selection agent. After PCR verification, agarose gel electrophoresis was performed to verify the transformants. Fifty-five transformants were proven to have the target sequence of the *cmcas9* gene because they showed obvious bands and were consistent with the expected size in an electrophoresis gel (Figure 3A). Five random transformants were tested by western blot, and the relevant size of the protein bands (186.5 kDa) was shown in the experimental samples, while the negative control was reversed (Figure 3B). The relatively high positive rate may be due to the successful selection mechanism of Basta and its resistance gene *blpR* as well as the modification of the ATMT protocol. One transformant chosen for fluorescence microscopy observation showed no obvious morphological change but significant fluorescence in cells (Figure 4), indicating that the Cas9-GFP fusion protein was successfully expressed. Then, qPCR was performed to test the transcription level of *cmcas9*. As shown in Figure 2B, *cmcas9* expressed a relatively high transcript level compared to the housekeeping gene *tef1*. The high transcript level may have been due to the strong efficiency of the gpd promotor and the codon optimized in *cas9*. This transformant also showed no significant difference in growth rate, indicating its availability for further study. Transformant *C. militaris:*: *blpR*-*cmcas9*-*gfp* was renamed as *C. militaris* C9. Since molecular expression elements were relatively rare in edible-medicinal mushrooms such as *C. militaris*, two new elements, the native promotor from the housekeeping gene *cmlsm3* and the terminator from *cmura3*, were newly discovered. Availability of the promoter Pcmgpd (Gong et al., 2009), which had been predicted to be usable in genome editing, and the Basta resistance gene *blpR* as a positive selection marker was also proved.

### Establishment of a CRISPR-Cas9 gene disruption system in *C. militaris*

Since the existence and function of sgRNA were relatively hard to detect, free sgRNA was chosen for the construction of the CRISPR-Cas9 system. To detect the efficiency of the CRISPR-Cas9 system, a target site was chosen in the *ura3* gene locus because URA3 will convert 5-FOA into a toxic compound and lead to cell death.

**Figure 5:**
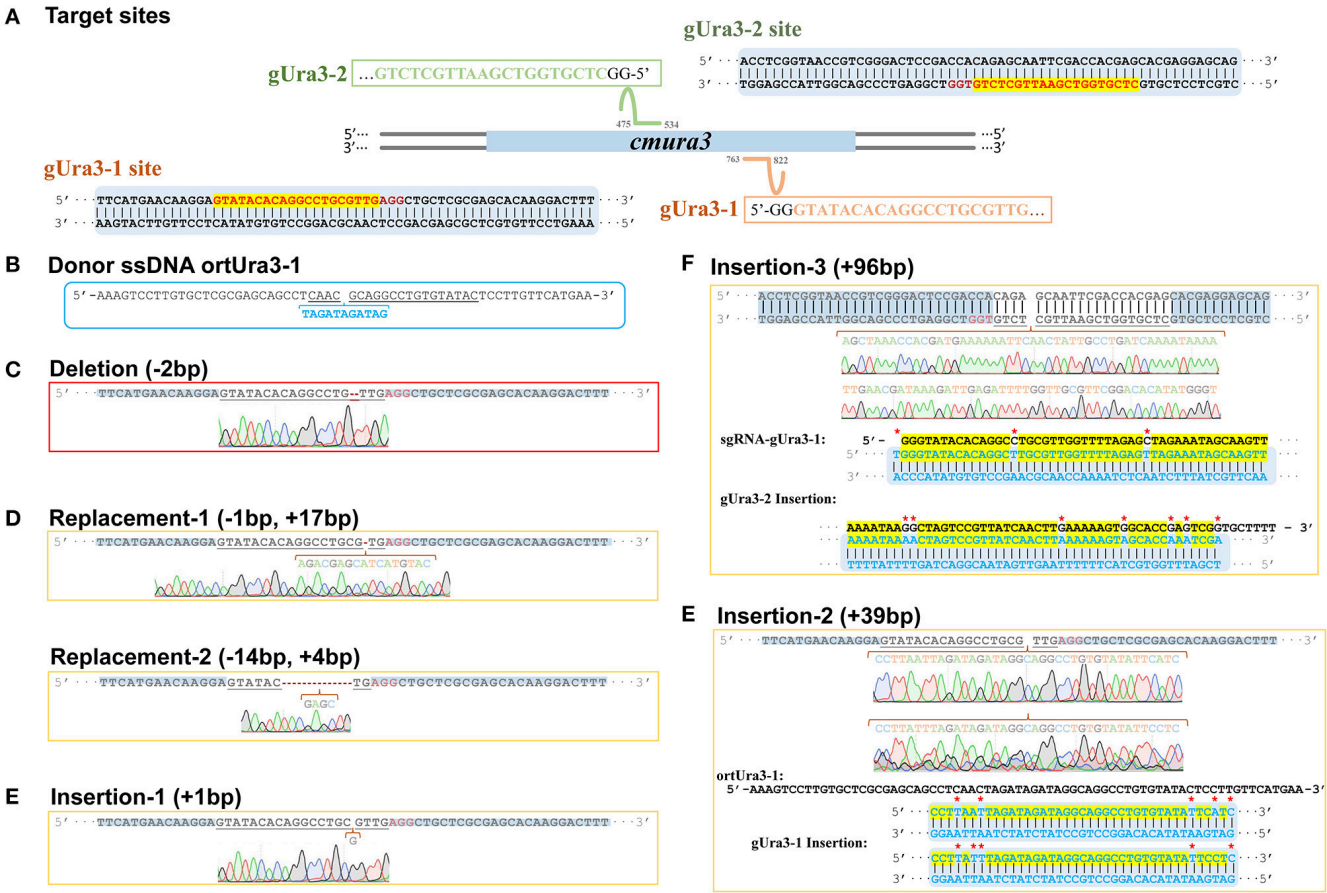
Sequence design and alignment diagrams for editing *cmura3* by CRISPR-Cas9 *in vivo*. **(A)** The localized sites and target sequences of sgRNA. (Apostrophe in the box represents the sgRNA scaffold sequence). **(B)** The sequence of donor ssDNA ortUra3-1. (The target sgRNA site was underlined; the inserted sequence was marked blue and listed separately). **(C)** The sequence and sequencing chromatogram for gUra3-1 deletion mutation. **(D)** The sequences and sequencing chromatograms for gUra3-1 replacement mutation. **(E)** The sequences and sequencing chromatograms for gUra3-1 insertion mutation and the alignments for the insertions. **(F)** The sequence and sequencing chromatogram for the gUra3-2 inserted mutation and alignment for the insertions.

As a negative control, the mycelia protoplasts of CM10 and C9 were treated with 5-FOA, and 30 and 22 individuals were presented, respectively. However, none of the *ura3* mutations was obtained while missing the relevant sgRNA. After transforming sgRNA-gUra3-1 and ortUra3-1 into mycelia protoplasts of *C. militaris*, we obtained a total of 51 individuals, which were grown out of the 5-FOA medium. Six mutant transformants of the gUra3-1 target site were obtained out of 51 putative transformants and are shown in Figures 5C–E (the sequencing chromatogram in the box shows the reliability of the sequencing results). As shown in Figure 5C, a 2-bp deletion (shown by the Red line) occurred in the gUra3-1 target (shown by the underlined sequence) and was located 4 bp upstream of the PAM site (AGG). Two kinds of replacement (1 bp replaced by 17 and 14 bp replaced by 4 bp) occurred and were located 3 bp upstream of the PAM site (Figure 5D). A 1-bp insertion occurred and was located 5 bp upstream of the PAM site (Figure 5E). It has been reported (Wiedenheft et al., 2012) that the 12 bp nearest the PAM site are the most important signal for sgRNA binding, so this mutation will abort the disruption by CRISPR-Cas9. Though we transformed the guide sgRNA along with their single-stranded repair templates, these 4 mutations showed that the target site was cut by Cas9 and repaired by NHEJ rather than HR. Interestingly, a special 39-bp insertion was also generated in the target site gUra3-1 and was located 4 bp upstream of the PAM site, and these 39 bp were similar (87.2%) to part of the homology repair template ortUra3-1 (71 bp), which showed that the double-stranded break may be repaired by a new mechanism other than NHEJ or HR in *C. militaris*.

All the disruption resulted in 5-FOA resistance (Boeke et al., 1984), proving the availability of orotidine 5′-phosphate decarboxylase (*ura3*) as a negative selection marker in *C. militaris*. Moreover, it was reported that mutations in URA3 will cause auxotrophy for uridine, but during the test, the URA3 mutants in *C. militaris* could still grow naturally in medium without adding uridine. This finding implied that the *ura3* gene was not as indispensable for cell growth in *C. militaris* as it is in *S. cerevisiae*.

For further testing of the efficiency of multidisruption, sgRNA-gUra3-1 and sgRNA-gUra3-2 were cotransformed into *C. militaris* C9 (CM10:: *blpR*-*cmcas9*-*gfp*) as well. Rather than the expected double-site mutation, mutations in the transformants showed that the gUra3-2 target site in the host had a 96-bp insert. As shown in Figure 5F, the insertion was located 5 bp upstream of the PAM site (TGG), and it was similar to sgRNA-gUra3-1 (84.3%), while the gUra3-1 site was not mutated.

## Discussion

In this research, a Cas9-expressing strain of *C. militaris* was constructed by using new-found molecular elements and ATMT. With transformation with an sgRNA that was simply synthesized *in vitro*, genome editing could be easy and flexible to apply in the traditional edible mushroom *C. militaris*.

Available selection markers are still lacking in medical ascomycetes fungi such as *C. militaris*, posing a real obstacle to the advancement of genome editing. Though high doses of Basta and 5-FOA could be used in this study, none of them could avoid high rates of false positives during the transformant selection. As a two-stage sac fungus, *C. militaris* is well-known for a high ratio of fruiting body gemmated degeneration in farming, which means a high dose of antifungal drugs might accelerate its degeneration to avoid the toxic effect.

Since gene integration leads to more stable expression in high-ratio degeneration sac fungus, the transformation of a single vector with an sgRNA-cas9 cassette was under consideration. A previous report (Gao and Zhao, 2014) showed that sgRNA could be generated by the RNA polymerase II promoter along with two specific ribozymes. These HH and HDV ribozymes have been used to generate sgRNA in plants, filamentous fungi (Mitchell et al., 2017) and even in the ascomycete fungus *Alternaria alternata* (Wenderoth et al., 2017). As previously reported, an sgRNA cassette was constructed with these two ribozymes and driven by a verified TrpC promotor and terminator. Since this strategy failed to edit the genome in *C. militaris*, qPCR was performed, which verified that the sgRNA was expressed successfully. It showed that the sgRNA cassette failed to generate functional sgRNA in this study because of the fail function of HH- or HDV-type ribozymes in *C. militaris*. Therefore, a strategy for generating a Cas9-expressing *C. militaris* strain and then transforming the presynthesized sgRNA has been performed.

The target sites gUra3-1 and gUra3-2 were located by transformed sgRNA-gUra3-1 or sgRNA-gUra3-2 and then identified by the editing protein Cas9-GFP. Subsequently, the DSBs were generated and repaired by the DNA repair mechanisms of *C. militaris*. There were six kinds of *ura3* mutations obtained on the 5-FOA plates while Cas9, sgRNA and/or donor DNA were present.

In the CRISPR-Cas9 editing system, double-stranded breaks caused by Cas9 will be generated near the PAM site and could be repaired by NHEJ or HR (Cong et al., 2013). In this study, all of the mutations that occurred were located 3-5 bp upstream of the PAM site of *ura3*, which was consistent with the Cas9 cleavage pattern. Therefore, we believed that the mutations were edited by the CRISPR system.

However, the mutation of three transformants showed an interesting repair mechanism. While transforming the sgRNA-Ura3-1 along with the corresponding micro oligonucleotide repair template ortUra3-1 into *C. militaris*, the expected specific site should have an insertion of the nucleotide sequence “TAGATAGATAG” which contains a terminator codon and would result in early termination while translating the mRNA of URA3. Microhomology-mediated end-joining has been used to repair the DSBs by mediating local sequence homology recombination according to the micro oligo templates in mammalian cells (Wang et al., 2013; Bae et al., 2014) and eukaryotic parasites (Peng et al., 2014). However, the unexpected 39-bp inserted results, which were DNA sequences similar to ortUra3-1 (Figure 5E), showed that the DSB was repaired by catching the free oligonucleotide fragment and pulling it into the break. In addition, while cotransforming two sgRNA into the cells, a 96-bp inserted sequence generated in gUra3-2 was similar to sgRNA-gUra3-1 (Figure 5F). One of the sgRNA nucleotides was accidentally caught and inserted into the DSB of the target site. These two kinds of mutations revealed that *C. militaris* has a unique repair mechanism that might repair DSBs by direct ligation or free nucleotide insertion. This random mismatch repair mechanism might be one of the reasons why *C. militaris* faces a high ratio of fruiting body gemmated degeneration.

In this study, we aimed to induce DSB repair by homology-directed repair but not random deletion or insertion. The single-stranded templates are more efficient than double-stranded in mammalian cells and some eukaryotic parasites (Peng and Tarleton, 2015), so the micro single-stranded oligonucleotides rather than double-stranded DNA were used as repair temples when utilizing the CRISPR system. However, it was hard to succeed with this microhomology-mediated end-joining strategy in *C. militaris*, which may due to the low homology recombination efficiency or other unknown reasons.

*C. militaris* is the primary species that can highly produce the valuable drug cordycepin. With this CRISPR technique, the pathway of cordycepin production will be the first target to edit. By disrupting the repressor gene or reconstructing the promotor of cordycepin synthetase, the yield of cordycepin will be further raised, and it will promote the development of the related industry.

To summarize, this is the first report of a successful CRISPR system development in the traditional edible-medicinal mushroom *C. militaris*, which could greatly raise hope for molecular breeding development to increase the production of bioactive strain degeneration delay.

## Author contributions

B-XC performed vector construction, RNA synthesis and was the major contributor in drafting the work; TW helped revise the manuscript critically for important intellectual content; H-BT performed strains transformation and PCR verification; FY and L-ZK performed strain cultivation; Z-WY supervised B-XC in sequencing data analysis; L-QG and J-FL agreed to be accountable for all aspects of the work in ensuring that questions related to the accuracy or integrity of any part of the work are appropriately investigated and resolved. All authors approved the last version of manuscript to be published.

### Conflict of interest statement

The authors declare that the research was conducted in the absence of any commercial or financial relationships that could be construed as a potential conflict of interest. The reviewer PP-N and handling Editor declared their shared affiliation.

## Abbreviations

sgRNA: single-guide RNA
NHEJ: non-homologous and joining
HR: homologous recombination
ssDNA: single-stranded DNA
*gpd*: gene for glycerol-3-phosphate dehydrogenase
*lsm3*: gene for U6 small nuclear ribonucleoprotein
*ura3*: gene for orotidine 5′-phosphate decarboxylase
ATMT: *Agrobacterium tumefaciens*-mediated transformation
qPCR: quantitative real-time PCR
gUra3: gRNA target sites of ura3
ortUra3: oligonucleotide repair templates for gUra3
5-FOA: 5-Fluoroorotic Acid.

## References

[B1] Bae S. Kweon J. Kim H. S. Kim J.-S. (2014). Microhomology-based choice of Cas9 nuclease target sites. Nat. Methods 11, 705–706. 10.1038/nmeth.3015 24972169

[B2] Binningerl D. M. Skrzynial C. Pukkila P. J. Casselton L. A. (1987). DNA-mediated transformation of the basidiomycete *Coprinus cinereus*. EMBO J. 6, 835–840. 3595558 10.1002/j.1460-2075.1987.tb04828.x PMC553472

[B3] Boeke J. D. Croute F. La Fink G. R. (1984). A positive selection for mutants lacking orotidine-5′-phosphate decarboxylase activity in yeast: 5-fluoro-orotic acid resistance. Mol. Gen. Genet. 197, 345–346. 10.1007/BF00330984 6394957

[B4] Chien C.-C. Tsai M.-L. Chen C.-C. Chang S.-J. Tseng C.-H. (2008). Effects on tyrosinase activity by the extracts of *Ganoderma lucidum* and related mushrooms. Mycopathologia 166, 117–120. 10.1007/s11046-008-9128-x 18459064

[B5] Cohen N. Cohen J. Asatiani M. D. Varshney V. K. Yu H.-T. Yang Y.-C.. (2014). Chemical composition and nutritional and medicinal value of fruit bodies and submerged cultured mycelia of culinary-medicinal higher basidiomycetes mushrooms. Int. J. Med. Mushrooms 16, 273–291. 10.1615/IntJMedMushr.v16.i3.80 24941169

[B6] Cong L. Ran F. A. Cox D. Lin S. Barretto R. Habib N.. (2013). Multiplex genome engineering using CRISPR/Cas systems. Science 339, 819–823. 10.1126/science.1231143 23287718 PMC3795411

[B7] Cui J. D. (2015). Biotechnological production and applications of *Cordyceps militaris*, a valued traditional Chinese medicine. Crit. Rev. Biotechnol. 35, 475–484. 10.3109/07388551.2014.900604 24666119

[B8] Gao Y. Zhao Y. (2014). Self-processing of ribozyme-flanked RNAs into guide RNAs *in vitro* and *in vivo* for CRISPR-mediated genome editing. J. Integr. Plant Biol. 56, 343–349. 10.1111/jipb.12152 24373158

[B9] Gong Z. Su Y. Huang L. Lin J. Tang K. Zhou X. (2009). Cloning and analysis of glyceraldehyde-3-phosphate dehydrogenase gene from *Cordyceps militaris*. African J. Agric. Res. 4, 402–408.

[B10] Katayama T. Tanaka Y. Okabe T. Nakamura H. Fujii W. Kitamoto K.. (2015). Development of a genome editing technique using the CRISPR/Cas9 system in the industrial filamentous fungus Aspergillus oryzae. Biotechnol. Lett. 38, 637–642. 10.1007/s10529-015-2015-x 26687199

[B11] Kim H. G. Shrestha B. Lim S. Y. Yoon D. H. Chang W. C. Shin D.-J.. (2006). Cordycepin inhibits lipopolysaccharide-induced inflammation by the suppression of NF-κB through Akt and p38 inhibition in RAW 264.7 macrophage cells. Eur. J. Pharmacol. 545, 192–199. 10.1016/j.ejphar.2006.06.047 16899239

[B12] Kistler K. E. Vosshall L. B. Matthews B. J. (2015). Genome engineering with CRISPR-Cas9 in the mosquito *Aedes aegypti*. Cell Rep. 11, 51-60. 10.1016/j.celrep.2015.03.009 25818303 PMC4394034

[B13] Lian T. Yang T. Liu G. Sun J. Dong C. C. Andersen C.. (2014). Reliable reference gene selection for *Cordyceps militaris* gene expression studies under different developmental stages and media. FEMS Microbiol. Lett. 356, 97–104. 10.1111/1574-6968.12492 24953133

[B14] Liu R. Chen L. Jiang Y. Zhou Z. Zou G. (2015). Efficient genome editing in filamentous fungus Trichoderma reesei using the CRISPR/Cas9 system. Cell Discov. 1:15007. 10.1038/celldisc.2015.7 27462408 PMC4860831

[B15] Livak K. J. Schmittgen T. D. (2001). Analysis of Relative Gene Expression Data Using Real-Time Quantitative, P. C. R., and the 2–ΔΔCT Method. Methods 25, 402–408. 10.1006/meth.2001.1262 11846609

[B16] Lou H. Ye Z. Yun F. Lin J. Guo L. Chen B.. (2018). Targeted gene deletion in *Cordyceps militaris* using the split-marker approach. Mol. Biotechnol. 60, 380–385. 10.1007/s12033-018-0080-9 29605840

[B17] Matsu-ura T. Baek M. Kwon J. Hong C. (2015). Efficient gene editing in *Neurospora crassa* with CRISPR technology. Fungal Biol. Biotechnol. 2:4. 10.1186/s40694-015-0015-1 28955455 PMC5611662

[B18] Mitchell A. P. Martinez D. Sakthikumar S. Anderson M. Berlin A. (2017). Location, location, location: use of CRISPR-Cas9 for genome editing in human pathogenic fungi. PLoS Pathog. 13:e1006209. 10.1371/journal.ppat.1006209 28358867 PMC5373618

[B19] Mullins E. D. Chen X. Romaine P. Raina R. Geiser D. M. Kang S. (2001). *Agrobacterium*-mediated transformation of *Fusarium oxysporum* : an efficient tool for insertional mutagenesis and gene transfer. Phytopathology 91, 173–180. 10.1094/PHYTO.2001.91.2.173 18944391

[B20] Nødvig C. S. Nielsen J. B. Kogle M. E. Mortensen U. H. (2015). A CRISPR-Cas9 system for genetic engineering of filamentous fungi. PLoS ONE 10:e0133085. 10.1371/journal.pone.0133085 26177455 PMC4503723

[B21] Noh E.-M. Kim J.-S. Hur H. Park B.-H. Song E.-K. Han M.-K. (2008). Cordycepin inhibits IL-1 -induced MMP-1 and MMP-3 expression in rheumatoid arthritis synovial fibroblasts. Rheumatology 48, 45–48. 10.1093/rheumatology/ken417 19056796

[B22] Peng D. Kurup S. P. Yao P. Y. Minning T. A. Tarleton R. L. (2014). CRISPR-Cas9-mediated single-gene and gene family disruption in Trypanosoma cruzi. MBio 6:e02097-14. 10.1128/mBio.02097-14 25550322 PMC4281920

[B23] Peng D. Tarleton R. (2015). EuPaGDT: a web tool tailored to design CRISPR guide RNAs for eukaryotic pathogens. Microb. Genomics 1, 1–7. 10.1099/mgen.0.000033 28348817 PMC5320623

[B24] Ray M. Tang R. Jiang Z. Rotello V. M. (2015). Quantitative tracking of protein trafficking to the nucleus using cytosolic protein delivery by nanoparticle-stabilized nanocapsules. Bioconjug. Chem. 26, 1004–1007. 10.1021/acs.bioconjchem.5b00141 26011555 PMC4743495

[B25] Reis F. S. Barros L. Calhelha R. C. Cirić A. van Griensven L. J. L. D. Soković M.. (2013). The methanolic extract of *Cordyceps militaris* (L.) Link fruiting body shows antioxidant, antibacterial, antifungal and antihuman tumor cell lines properties. Food Chem. Toxicol. 62, 91–98. 10.1016/j.fct.2013.08.033 23994083

[B26] Taofi O. González-Paramás A. M. Martins A. Barreiro M. F. Ferreira I. C. F. R. (2016). Mushrooms extracts and compounds in cosmetics, cosmeceuticals and nutricosmetics—a review. Ind. Crops Prod. 90, 38–48. 10.1016/j.indcrop.2016.06.012

[B27] Tuli H. S. Kashyap D. Sharma A. K. (2015). Cordycepin: a cordyceps metabolite with promising therapeutic potential, in Fungal Metabolites, eds Mérillon J.-M. Ramawat K. G. (Cham: Springer International Publishing), 1–22.

[B28] Wang H. Yang H. Shivalila C. S. Dawlaty M. M. Cheng A. W. Zhang F.. (2013). One-step generation of mice carrying mutations in multiple genes by CRISPR/Cas-mediated genome engineering. Cell 153, 910–918. 10.1016/j.cell.2013.04.025 23643243 PMC3969854

[B29] Wang L. Zhang W. M. Hu B. Chen Y. Q. Qu L. H. (2008). Genetic variation of *Cordyceps militaris* and its allies based on phylogenetic analysis of rDNA ITS sequence data. Fungal Divers. 31, 147–155.

[B30] Wenderoth M. Pinecker C. Voß B. Fischer R. (2017). Establishment of CRISPR/Cas9 in *Alternaria alternata*. Fungal Genet. Biol. 101, 55–60. 10.1016/j.fgb.2017.03.001 28286319

[B31] Wiedenheft B. Sternberg S. H. Doudna J. A. (2012). RNA-guided genetic silencing systems in bacteria and archaea. Nature 482, 331–338. 10.1038/nature10886 22337052

[B32] Wu W.-C. Hsiao J.-R. Lian Y.-Y. Lin C.-Y. Huang B.-M. (2007). The apoptotic effect of cordycepin on human OEC-M1 oral cancer cell line. Cancer Chemother. Pharmacol. 60, 103–111. 10.1007/s00280-006-0354-y 17031645

[B33] Yang T. Guo M. Yang H. Guo S. Dong C. (2016). The blue-light receptor CmWC-1 mediates fruit body development and secondary metabolism in *Cordyceps militaris*. Appl. Microbiol. Biotechnol. 100, 743–755. 10.1007/s00253-015-7047-6 26476643

[B34] Zhang G. Yin Q. Han T. Zhao Y. Su J. Li M. (2015). Purification and antioxidant effect of novel fungal polysaccharides from the stroma of *Cordyceps kyushuensis*. Ind. Crops Prod. 69, 485–491. 10.1016/j.indcrop.2015.03.006

[B35] Zheng P. Xia Y. Xiao G. Xiong C. Hu X. Zhang S.. (2011). Genome sequence of the insect pathogenic fungus *Cordyceps militaris*, a valued traditional chinese medicine. Genome Biol. 12:R116. 10.1186/gb-2011-12-11-r116 22112802 PMC3334602

[B36] Zheng Z. Huang C. Cao L. Xie C. Han R. (2011). *Agrobacterium tumefaciens*-mediated transformation as a tool for insertional mutagenesis in medicinal fungus *Cordyceps militaris*. Fungal Biol. 115, 265–274. 10.1016/j.funbio.2010.12.011 21354533

[B37] Zhou X. Cai G. He Y. Tong G. (2016). Separation of cordycepin from *Cordyceps militaris* fermentation supernatant using preparative HPLC and evaluation of its antibacterial activity as an NAD+-dependent DNA ligase inhibitor. Exp. Ther. Med. 12, 1812–1816. 10.3892/etm.2016.3536 27588098 PMC4998010

