# Supplementary material for: Efficient CRISPR-Cas9 Gene Disruption System in Edible-Medicinal Mushroom Cordyceps militaris
Source: Front Microbiol. 2018 Jun 12;9:1157. doi: 10.3389/fmicb.2018.01157 (PMC6005869; doi:10.3389/fmicb.2018.01157)
Supplement: Supplementary file 1 [file Table_1.DOCX]

Supplementary Material

Efficient CRISPR-Cas9 gene disruption system in traditional edible mushroom *Cordyceps militaris*

Bai-Xiong Chen*

*** Correspondence:** Li-Qiong Guo: guolq@scau.edu.cn; Jun-Fang Lin: linjf@scau.edu.cn

# Supplementary Tables

Supplementary Table S1-6: Table S1. Sequence of codon-optimized cas9 for *Cordyceps militaris* (CmCas9_eGFP_C-Myc). Table S2. Sequence of promotor from U6 small nuclear ribonucleoprotein (*lsm3*) in *C. militaris* (CCM_07996, accession No. NW_006271974.1) (Pcmlsm3). Table S3. Sequence of terminator from orotidine 5'-phosphate decarboxylase (*ura3*) in *C. militaris* (CCM_05256, accession No. NW_006271972.1) (Tcmura3). Table S4. Sequence of donor single-stranded DNA (ssDNA) template ortUra3-1. Table S5. Sequence of sgRNA sgRNA-gUra3-1 and sgRNA-gUra3-2. Table S6. Sequence of sgRNA cassette (Promoter from *Aspergillus nidulans* trpC (PtrpC), hammerhead type ribozymes, Ura3-1_gRNA scaffold, hepatitis delta virus type ribozymes and terminator from *Aspergillus nidulans* trpC (TtrpC).

## Table S1. Sequence of codon-optimized *cas9* for *Cordyceps militaris*.

**CmCas9_eGFP_C-Myc:**

5’-ATGGACAAGAAGTACTCTATCGGCCTTGACATTGGTACAAACAGCGTTGGTTGGGCTGTTATCACAGACGAGTACAAGGTCCCATCTAAGAAGTTCAAGGTCCTGGGTAACACAGACCGTCACTCCATTAAGAAGAACCTTATTGGTGCCCTGCTTTTCGACTCGGGTGAGACAGCCGAGGCCACACGTCTGAAGCGCACCGCTCGTCGCCGATACACCCGCCGAAAGAACCGAATTTGCTACCTCCAAGAGATTTTCTCTAACGAGATGGCTAAGGTTGACGACTCTTTCTTCCACCGTCTTGAAGAGTCTTTCCTGGTCGAAGAGGACAAGAAGCACGAGCGCCACCCTATTTTCGGCAACATCGTTGACGAGGTCGCCTACCACGAGAAGTACCCTACCATATACCACCTGCGAAAGAAGCTTGTCGACTCTACAGACAAGGCTGACCTGCGCCTGATATATCTCGCTCTTGCCCACATGATCAAGTTCCGTGGTCACTTCCTTATTGAGGGCGACCTCAACCCCGACAACTCGGACGTTGACAAGCTTTTCATTCAGCTCGTTCAGACATACAACCAGCTTTTCGAAGAAAACCCAATTAACGCTAGCGGTGTCGACGCCAAGGCTATTCTGTCGGCCCGCCTGTCCAAGTCGCGCCGCCTGGAGAACCTCATCGCTCAGCTCCCCGGTGAGAAGAAGAACGGTCTGTTCGGCAACCTTATTGCCCTTAGCCTCGGCCTCACCCCAAACTTCAAGTCTAACTTCGACCTTGCCGAGGACGCTAAGCTCCAGCTGTCTAAGGACACCTACGACGACGACCTTGACAACCTCCTTGCTCAGATTGGTGACCAGTACGCTGACCTGTTCCTTGCCGCTAAGAACCTCTCGGACGCCATTCTGCTTAGCGACATTCTGCGTGTCAACACAGAGATCACAAAGGCCCCCCTCTCGGCTAGCATGATTAAGCGATACGACGAGCACCACCAGGACCTTACACTGCTCAAGGCTCTTGTCCGTCAGCAGCTGCCAGAGAAGTACAAGGAAATTTTCTTCGACCAGTCTAAGAACGGTTACGCCGGTTACATTGACGGCGGTGCCTCGCAGGAAGAGTTCTACAAGTTCATTAAGCCAATTCTTGAGAAGATGGACGGCACAGAAGAACTGCTTGTTAAGCTGAACCGTGAGGACCTCCTGCGAAAGCAGCGCACATTCGACAACGGTAGCATCCCTCACCAGATCCACCTTGGTGAGCTGCACGCTATTCTGCGCCGTCAAGAAGACTTCTACCCATTCCTTAAGGACAACCGCGAGAAGATTGAGAAGATTCTTACATTCCGAATCCCATACTACGTTGGCCCACTGGCTCGTGGCAACTCGCGATTCGCTTGGATGACCCGAAAGTCGGAAGAAACCATTACCCCTTGGAACTTCGAAGAAGTTGTCGACAAGGGTGCCTCGGCCCAGTCTTTCATTGAGCGTATGACCAACTTCGACAAGAACCTCCCAAACGAGAAGGTCCTCCCAAAGCACTCTCTGCTGTACGAGTACTTCACAGTTTACAACGAGCTTACAAAGGTTAAATACGTTACAGAGGGTATGCGAAAGCCCGCTTTCCTGTCGGGCGAGCAGAAGAAGGCTATCGTTGACCTGCTGTTCAAGACCAACCGAAAGGTTACAGTTAAGCAGCTTAAGGAAGACTACTTCAAGAAGATTGAGTGCTTCGACTCCGTTGAGATCTCGGGCGTTGAGGACCGATTCAACGCTAGCCTGGGCACCTACCACGACCTGCTTAAGATTATTAAGGACAAGGACTTCCTGGACAACGAGGAAAACGAGGACATTCTGGAGGACATCGTTCTCACACTTACACTTTTCGAGGACCGTGAGATGATCGAGGAACGTCTGAAGACATACGCTCACCTTTTCGACGACAAGGTTATGAAGCAGCTGAAGCGTCGCCGATACACCGGTTGGGGCCGTCTGTCCCGAAAGCTCATCAACGGCATCCGTGACAAGCAGTCGGGCAAGACCATTCTTGACTTCCTGAAGTCCGACGGTTTCGCTAACCGCAACTTCATGCAGCTTATTCACGACGACTCTCTTACATTCAAGGAAGACATCCAGAAGGCTCAGGTTTCCGGTCAGGGTGACTCTCTGCACGAGCACATCGCTAACCTGGCCGGTAGCCCCGCTATCAAGAAGGGTATCCTGCAGACAGTTAAGGTTGTTGACGAGCTTGTTAAGGTTATGGGCCGCCACAAGCCAGAGAACATTGTTATTGAGATGGCCCGTGAGAACCAGACCACACAGAAGGGTCAGAAGAACTCGCGCGAGCGCATGAAGCGAATTGAGGAAGGTATTAAAGAACTGGGTAGCCAGATCCTTAAAGAACACCCAGTTGAGAACACCCAGCTGCAGAACGAGAAGCTTTACCTTTACTACCTGCAGAACGGTCGTGACATGTATGTTGACCAGGAACTGGACATTAACCGCCTGTCGGACTACGACGTTGACCACATCGTCCCTCAGTCTTTCCTGAAGGACGACTCTATCGACAACAAGGTCCTCACACGTAGCGACAAGAACCGCGGCAAGTCTGACAACGTTCCATCTGAGGAAGTCGTTAAGAAGATGAAGAACTACTGGCGTCAGCTGCTTAACGCTAAGCTTATCACCCAGCGGAAGTTCGACAACCTCACAAAGGCTGAGCGTGGTGGTCTGTCGGAGCTGGACAAGGCCGGTTTCATTAAGCGTCAGCTTGTTGAGACCCGTCAGATCACAAAGCACGTCGCTCAGATTCTGGACTCCCGCATGAACACCAAGTACGACGAGAACGACAAGCTCATCCGTGAGGTCAAGGTTATTACACTGAAGTCTAAGCTTGTCTCGGACTTCCGAAAGGACTTCCAGTTCTACAAGGTCCGCGAGATTAACAACTACCACCACGCTCACGACGCCTACCTTAACGCTGTTGTTGGCACAGCCCTGATCAAGAAGTACCCAAAGCTGGAGTCTGAGTTCGTTTACGGTGACTACAAGGTTTACGACGTCCGTAAGATGATCGCTAAGTCGGAGCAGGAAATTGGCAAGGCCACCGCTAAGTACTTCTTCTACTCTAACATCATGAACTTCTTCAAGACAGAGATCACACTGGCTAACGGTGAGATCCGAAAGCGTCCCCTTATCGAGACCAACGGCGAGACCGGTGAGATTGTTTGGGACAAGGGCCGTGACTTCGCCACAGTTCGTAAGGTCCTGTCCATGCCTCAGGTCAACATCGTCAAGAAGACAGAGGTTCAGACCGGCGGTTTCTCCAAAGAGTCGATTCTCCCTAAGCGTAACTCGGACAAGCTTATTGCCCGAAAGAAGGACTGGGACCCCAAGAAGTACGGCGGTTTCGACTCCCCTACCGTTGCCTACTCCGTCCTCGTTGTTGCTAAGGTCGAGAAGGGTAAATCTAAGAAGCTGAAGTCTGTTAAAGAACTCCTGGGTATCACCATCATGGAGCGATCGTCTTTCGAGAAGAACCCTATCGACTTCCTGGAGGCTAAGGGCTACAAAGAAGTCAAGAAGGACCTGATCATTAAGCTCCCAAAGTACTCTCTGTTCGAGCTGGAGAACGGCCGAAAGCGTATGCTTGCTAGCGCCGGTGAGCTGCAGAAGGGTAACGAGCTTGCCCTCCCTAGCAAATATGTTAACTTCCTGTACCTTGCCTCGCACTACGAGAAGCTGAAGGGTAGCCCAGAGGACAACGAGCAGAAGCAGCTTTTCGTTGAGCAGCACAAGCACTACCTGGACGAGATTATTGAGCAGATCTCGGAGTTCTCTAAGCGCGTCATCCTTGCCGACGCCAACCTTGACAAGGTTCTCTCGGCCTACAACAAGCACCGCGACAAGCCAATCCGAGAGCAGGCTGAGAACATTATCCACCTGTTCACACTCACAAACCTTGGTGCCCCCGCTGCTTTCAAGTACTTCGACACCACAATTGACCGTAAGCGATACACATCCACAAAAGAAGTTCTGGACGCCACACTGATCCACCAGTCTATCACAGGCCTTTACGAGACCCGAATTGACCTTAGCCAGCTGGGCGGTGAC (*cmcas9*)

GAGGGTGCC

GTGAGCAAGGGCGAGGAGCTGTTCACCGGGGTGGTGCCCATCCTGGTCGAGCTGGACGGCGACGTAAACGGCCACAAGTTCAGCGTGTCCGGCGAGGGCGAGGGCGATGCCACCTACGGCAAGCTGACCCTGAAGTTCATCTGCAcCACCGGCAAGCTGCCCGTGCCCTGGcCCACCCTCGTGACCACCCTGACCTACGGCGTGCAGTGCTTCAGCCGCTACCCCGACcACATGAAGCAGCACGACTTCTTCAAGTCCGCCATGCCCGAAGGCTACGTCCAGGAGCGCACCATCTTCTTCAAGGACGACGGCAACTACAAGACCCGCGCCGAGGTGAAGTTCGAGGGCGACACCCTGGTGAACCGCATCGAGCTGAAGGGCATCGACTTCAAGGAGGACGGCAACATCCTGGGGCACAAGCTGGAGTACAACTACAACAGCCACAACGTCTATATCATGGCCGACAAGCAGAAGAACGGCATCAAGGTGAACTTCAAGATCCGCCACAACATCGAGGACGGCAGCGTGCAGCTCGCCGACCACTACCAGCAGAACACCCCCATCGGCGACGGCCCCGTGCTGCTGCCCGACAACCACTACCTGAGCACCCAGTCCGCCCTGAGCAAAGACCCCAACGAGAAGCGCGATCACATGGTCCTGCTGGAGTTCGTGACCGCCGCCGGGATCACTCTCGGCATGGACGAGCTGTACAAG (gfp)

CCTGCCGCCAAGCGTGTCAAGCTTGAC (*myc*)

TAA -3’

## Table S2. Sequence of promotor from U6 small nuclear ribonucleoprotein (lsm3) in *C. militaris* (CCM_07996, accession No. NW_006271974.1) (Pcmlsm3)

5’-TGTCTTTTCGCGCGGCGGCTGCACATCAGCAAACTGTGGCCGAATGATGGGTGTTTCTGCGTGCGTGGCTAGGCATCGTCGCCCCAGCCGAGTCGCTGCTCGCGTGTAGCCTGTGGTGTGCTGCGTCTTGGCGACGAGCGCGGAGCGACGACCGAGGCATGCTATTGCCAACGTCGGTGACCGCGAGCCCATCTCTGATCAGAGCCCAGCAGCTTTGGCGGAATATTACCGCTCAATTGGACTCGTGGGCTCTAAATCGCAGCGTTCGCTCGATAATAGATGAGGAGGACCTGGGGGCGGGACGGGTGGATCTGGTCTGAGGGCAAGTTGGGGTAAACGGGGCCACAACCGAGCGGCTGGTTAGTTGCATATGTCTGCTGTGCTCCGATTAATGCATAATTCGCCCGAAGATTTTCCAGCTATGACCTGAGATAGCTCAACCCTTGCAACATCGCAGCTGGTATTCGCGAACCCGCCACACGCCACTCATTCATCACAAAATTGATCTAGCGGACTTGCTTCACAACAGAGCGCTCATAATTTCATA -3’

## Table S3. Sequence of terminator from orotidine 5'-phosphate decarboxylase (ura3) in C. militaris (CCM_05256, accession No. NW_006271972.1) (Tcmura3)

5’-TGGCACCACTTCGTCTTCATTTGTTTTTTTTGCATATACTTGGCTTGCTTTGATTATGGTTATTTTGGGCTATGCTGTTTTTCTTTCCCTTACCTTTTACGATGTCGTATGAGTGAACTTGCTTGGAATGGATTTATCTTTTTTTCTTTTTTTCTTTTCTTTTTTTTTTGGTTGCCTCGTCTGCTTCTTCATACCCTCTTCGCCTGAAGATGAGAAAAAGTTGTTGCCAAGCGTGGAGCACTGGAAGGAAAAGCATCGTGATGCGCTTGACAGATGAAATGAGATGAACGAGATTGAAAGAGAACATGATTTTCAACTTTGAGATCCAACGCCCATTCGCATCGTCATTGTTGGCTCGGCCATTTGTTCAGTAGAAACTGTTGTATAATCCGAGTTCATGATGATATATCAATAATCTAGGCCGTGAGTCAAGCGGCTGTTTGTTCGACCCACCCATTGCGCGGACTTAACGGCCCGTAGACGTCCCTGATAACCTTTTTGA -3’

## Table S4. Sequence of donor single-stranded DNA (ssDNA) template

**ortUra3-1:**

5’-AAAGTCCTTGTGCTCGCGAGCAGCCTCAACTAGATAGATAGGCAGGCCTGTGTATACTCCTTGTTCATGAA -3’

## Table S5. Sequence of sgRNA

sgRNA-gUra3-1:

5’-GG**GTATACACAGGCCTGCGTTG**GTTTTAGAGCTAGAAATAGCAAGTTAAAATAAGGCTAGTCCGTTATCAACTTGAAAAAGTGGCACCGAGTCGGTGCTTTT -3’

sgRNA-gUra3-2:

5’-GG**CTCGTGGTCGAATTGCTCTG**GTTTTAGAGCTAGAAATAGCAAGTTAAAATAAGGCTAGTCCGTTATCAACTTGAAAAAGTGGCACCGAGTCGGTGCTTTT -3’

## Table S6. Sequence of sgRNA cassette.

Promoter from *Aspergillus nidulans* trpC (PtrpC)

Hammerhead type ribozymes (HH)

Ura3-1_gRNA scaffold

Hepatitis delta virus type ribozymes (HDV)

Terminator from *Aspergillus nidulans* trpC (TtrpC)

5’- cctagg (*BlnI*)

CGCGCAAGGATCCTCTAGATCTCGAGGCCTGATCATCGATGGGCCCATCGATGATCAGGCCTCGACAGAAGATGATATTGAAGGAGCACTTTTTGGGCTTGGCTGGAGCTAGTGGAGGTCAACAATGAATGCCTATTTTGGTTTAGTCGTCCAGGCGGTGAGCACAAAATTTGTGTCGTTTGACAAGATGGTTCATTTAGGCAACTGGTCAGATCAGCCCCACTTGTAGCAGTAGCGGCGGCGCTCGAAGTGTGACTCTTATTAGCAGACAGGAACGAGGACATTATTATCATCTGCTGCTTGGTGCACGATAACTTGGTGCGTTTGTCAAGCAAGGTAAGTGAACGACCCGGTCATACCTTCTTAAGTTCGCCCTTCCTCCCTTTATTTCAGATTCAATCTGACTTACCTATTCTACCCAAGCATCGAT (PtrpC)

tctaga (*XbaI*)

GTATACCTGATGAGTCCGTGAGGACGAAACGAGTAAGCTCGTC (HH)

GTATACACAGGCCTGCGTTG (gUra3-1)

GTTTTAGAGCTAGAAATAGCAAGTTAAAATAAGGCTAGTCCGTTATCAACTTGAAAAAGTGGCACCGAGTCGGTGGTGCTTTT (gRNA scaffold)

GGCCGGCATGGTCCCAGCCTCCTCGCTGGCGCCGGCTGGGCAACATGCTTCGGCATGGCGAATGGGAC (HDV)

tctaga (*XbaI*)

TGATTTAATAGCTCCATGTCAACAAGAATAAAACGCGTTTCGGGTTTACCTCTTCCAGATACAGCTCATCTGCAATGCATTAATGCATTGGACCTCGCAACCCTAGTACGCCCTTCAGGCTCCGGCGAAGCAGAAGAATAGCTTAGCAGAGTCTATTTTCATTTTCGGGAGACGAGATCAAGCAGATCAACGGTCGTCAAGAGACCTACGAGACTGAGGAATCCGCTCTTGGCTCCACGCGACTATATATTTGTCTCTAATTGTACTTTGACATGCTCCTCTTCTTTACTCTGATAGCTTGACTATGAAAATTCCGTCACCAGCCCCTGGGTTCGCAAAGATAATTGCACTGTTTCTTCCTTGAACTCTCAAGCCTACAGGACACACATTCATCGTAGGTATAAACCTCGAAAATCATTCCTACTAAGATGGGTATACAATAGTAACCATGGTTGCCTAGTGAATGCTCCGTAACACCCAATACGCCGGCCGAAACTTTTTTACAACTCTCCTATGAGTCGTTTACCCAGAATGCACAGGTACACTTGTTTAGAGGTAATCCTTCTTTCTAG (TtrpC)

gaattc (*EcoRI*) -3’
